# Evaluation of Electrical Characteristics of Weft-Knitted Strain Sensors for Joint Motion Monitoring: Focus on Plating Stitch Structure

**DOI:** 10.3390/s24237581

**Published:** 2024-11-27

**Authors:** You-Kyung Oh, Youn-Hee Kim

**Affiliations:** Department of Convergence Design and Technology, Kookmin University, Seoul 02707, Republic of Korea; fbdk96@kookmin.ac.kr

**Keywords:** knitted strain sensors, plain stitch, plating stitch structure, stitch pattern, yarn thickness, NP number, joint motion monitoring

## Abstract

We developed a sensor optimized for joint motion monitoring by exploring the effects of the stitch pattern, yarn thickness, and NP number on the performance of knitted strain sensors. We conducted stretching experiments with basic weft-knit patterns to select the optimal stitch pattern and analyze its sensitivity and reproducibility. The plain stitch with a conductive yarn located on the reverse side exhibited the highest gauge factor value (143.68) and achieved excellent performance, with a stable change in resistance even after repeated sensing. For an in-depth analysis, we developed six sensors using the aforementioned pattern with different combinations of yarn thickness (1-ply, 2-ply) and NP numbers (12, 13, 14). Based on bending experiments, the GF across all sensors was 60.2–1092, indicating noticeable differences in sensitivity. However, no significant differences were observed in reproducibility, reliability, and responsiveness, confirming that all the sensors are capable of joint motion monitoring. Therefore, the plain-patterned plating stitch structure with conductive yarn on the reverse side is optimal for joint motion monitoring, and the yarn thickness and NP numbers can be adjusted to suit different purposes. This study provides basic data for developing knitted strain sensors and offers insights into how knitting methods impact sensor performance.

## 1. Introduction

Smart wearables provide users with an advanced experience by integrating functions [1,2,3] such as sensing [4,5], energy harvesting [6,7], energy storage [8,9], antenna [10,11], and actuator [12,13]. Stretchable devices are a core material for smart wearables and are being developed to measure temperature, pressure, and movement at various locations on the body [14,15,16]. Such sensors are in direct contact with the body and periodically measure biosignal information to provide appropriate feedback to users based on the collected information. Therefore, the development of textronic-based sensors should focus on ensuring an appropriate wearing sensation because such sensors must be able to collect data continuously without causing any inconvenience to the wearer. Textronics are developed by coating and impregnating conductive materials into fabrics or through embroidering and knitting using conductive yarns to enable the fibers themselves to perform sensing. Among these, knitted strain sensors remain in close contact with the body, which facilitates data collection, and are comfortable to wear, thereby being ideal for joint monitoring. Such sensors possess a loop-type network of yarn and function based on the principle that resistance increases or decreases as the contact point between the conductive yarns changes with stretching and recovery, respectively [17]. Several studies have utilized knitted strain sensors to measure movement by placing them in joint areas such as fingers, wrists, elbows, knees, and feet, where deformation occurs [18,19,20,21,22,23,24]. For example, Li et al. [25] studied the resistance response based on sensor position to monitor the movement of knee joints. Liang et al. [26] investigated the impact of wales, course count, and spandex content on the size of knitted strain sensors and analyzed their performance in measurements of wrist, elbow, neck, and finger joints. Bozali et al. [27] selected different combinations of elastomeric and non-elastomeric yarn to monitor the movement of fingers and wrists, comparing the sensing properties based on the material used. As such, previous studies have examined the dependence of performance on the size, position, and material of the sensor to develop sensors optimized for joints. However, such factors are not likely to fundamentally influence the sensor’s performance. Therefore, the study variables must be selected based on a clear comprehension of the distinctive characteristics and detection mechanisms of knitted strain sensors.

Therefore, our research focuses on loops, which constitute a foundational element of the knit structure. We investigate the impact of knitting techniques, including stitch patterns, yarn thickness, and needle position (NP) number, on sensor performance to develop sensors optimized for monitoring joint motion. Initially, we create three basic stitch patterns to assess resistance changes during stretching and evaluate how the loop connection method affects sensor performance. We also explore the impact of loop characteristics on the number of contact points through sensor surface photographs and 3D modeling; accordingly, the best stitch patterns are selected. For a more detailed analysis, we create six sensors with different yarn thickness and NP numbers and study the effect of loop width and loop length on sensor performance. Additionally, to evaluate the suitability for motion monitoring, we conduct bending experiments with different angles and bending rates and analyze electrical properties such as sensitivity, reproducibility, reliability, and responsiveness. Based on the sensing mechanism, we comprehensively examine the changes in sensor performance depending on the knitting method and propose a knitted strain sensor structure optimized for joint motion monitoring. Our findings can be used as baseline data for the development of knitted strain sensors tailored to different joint areas and purposes. This highlights the potential applicability of knitted strain sensors in the smart fashion industry.

## 2. Materials and Methods

### 2.1. Sensor Material

To understand the impact of structural deformation on electrical resistance, we used C&TEX’s (Seoul, Republic of Korea) nonconductive yarns, consisting of non-stretchable acryl and wool in a 1:1 ratio. To ensure that the resistance of the fiber strain gauge would be affected only by shape deformation, we selected a silver-coated polymer yarn with a uniform surface as a conductive yarn [28]. The conductive yarn that served as the sensor was a polyamide/polyester from AMANN (Bonnigheim, Germany), the high resistance (<530 Ω/m) of which helped maximize the change in the sensor’s resistance when stretched. Finally, the conductive yarn that acted as the wire for the microcontroller unit (MCU) was a low-resistance (<85 Ω/m) silver-coated yarn from AMANN (Bonnigheim, Germany), which was selected considering the thickness required for compatibility with a sewing machine [4].

### 2.2. Design and Fabrication Methods

**Figure 1:**
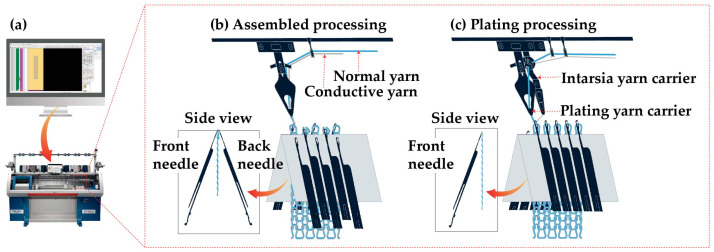
Pattern design and knitting process for knitted strain sensor: (**a**) pattern software and computerized flat knitting machine; (**b**) methods of knitting purl and rib stitches; and (**c**) plating stitch structure for knitting plain pattern.

Figure 1a illustrates the pattern-making and knitting process. The sensor was designed by adjusting the variables using the M1 Plus 7.2.037 pattern software, and it was knitted using a CMS330 KI W TT SPORT E7.2 (14 gauge) computerized flat knitting machine (STOLL, Reutingen, Germany). We set the sensor size to 30 mm × 150 mm with reference to Lee et al., who measured the length of the knee body surface during a 120° operation [29]. The design of the sensor was based on three variables, namely stitch pattern, yarn thickness, and NP number, which represent the effects of the sensor’s loop connection method, length, and width on its performance. The stitch pattern comprised three basic patterns of weft-knitting fabric and was categorized as plain stitch, purl stitch, or rib stitch, depending on how the loop was connected [30]. We used the optimal knitting method for each stitch pattern, and nonconductive yarn (sky blue) and conductive yarn (gray) were used for sensor fabrication. Figure 1b shows the purl stitch and rib stitch knitting method, in which the nonconductive and conductive yarns were assembled and fed to the plating yarn carrier and knitted using front and back needles. The purl and rib stitches had the same structure on their front and reverse sides and were designed as 1 × 1 structure. However, because the plain stitch had a clear difference between the front and reverse sides, the conductive yarns were evenly placed on the front or reverse side using a plating knitting technique. The plating stitch structure represents a technique for simultaneously knitting two or more yarns to create different colors, textures, and other effects on the surface of a fabric. Thus, by adjusting the yarn carrier position, we could determine the position of the conductive yarn; accordingly, positioning the yarn closest to the needle hook allowed it to be knitted on the reverse side of the fabric [31]. As shown in Figure 1c, we used a plating yarn carrier and an intarsia yarn carrier to supply the yarns individually to the needle hook and knitted them using the front needle. This provides a foundation for in-depth research to determine how the loop connection structure affects the number of contacts. Yarn thickness refers to the number of yarns used in knitting. As more yarns are added, the width of the loop decreases, increasing density and reducing strain. While a 1-ply was consistently used for the conductive yarns, the knitted sensor had either a 1-ply or 2-ply based on the addition of nonconductive yarn. This enabled us to determine how density—which depends on the loop width, number of contacts, and contact pressure)—affects the sensor’s performance. The NP number is related to the size and length of the loop. The knit density is determined by the NP number—the higher the number, the lower the density of the knit fabric, and the lower the number, the higher the density of the knit fabric [32]. Moreover, a wider lower loop and a longer higher loop permit the sensor to exhibit higher strain [33]. Accordingly, the effect of the density and strain of the knitted fabric—which are linked to the NP number—on the number of contacts and contact pressure can be determined.

### 2.3. Analysis Method

Monitoring joint movement requires a highly sensitive sensor because a wide range of movements, from small to large, must be detectable. The gauge factor (GF) is a vital parameter related to sensitivity [15]. It represents the slope of the relative change in resistance to applied strain; thus, it should be maximized to ensure that the sensor is as sensitive as possible [15]. The knitted strain sensors used in our study showed a negative piezo effect, whereby resistance decreases with stretching. Thus, the initial resistance is the maximum value, and the resistance after stretching is the minimum value [4]. We calculated GF by substituting the minimum value into the initial resistance ($R_{0}$) term in Equation (1); this equation was formulated by Jang et al. [34], who investigated strain sensors exhibiting the same resistance change pattern.

(1) $$GF=\frac{\frac{∆R}{R_{0}}}{\frac{∆L}{L_{0}}}=\frac{∆R}{\varepsilon R_{0}}$$

$R_{0}$: Initial resistance → After stretching (min resistance);

∆R: Applied resistance;

$L_{0}$: Initial length;

∆L: Applied length;

ε: Strain value.

## 3. Experimental Section

We conducted stretching tests and selected the best stitch pattern to determine the effect of stitch patterns on contact resistance. Furthermore, we conducted bending tests with various angles and bending rates by using the yarn thickness and NP number as variables for the selected stitch patterns to optimize the sensor’s performance in monitoring joint motion.

### 3.1. Experiment Preparation

Before starting the experiment, we added a lockstitch of conductive yarn to the sensor that acted as a wire for measuring the resistance change of the sensor. The lockstitch was designed in a zigzag form with 3 mm intervals in consideration of the elasticity of the knit to prevent the conductive yarn from breaking [4]. We also added a snap fastener acting as a ground (GND) connection when connecting the sensor to the hardware. In addition, we performed pre-stretching five times per sample to continuously output the same electrical signal from the strain sensor [35].

### 3.2. Stretching Test

We examined the change in the sensor’s resistance to strain and evaluated its sensitivity and reproducibility by stretching the plain, purl, and rib stitches. The sensor was tested 10 times with a maximum stretch of 30%. A pair of 50 mm clips was used to fix the fabric in the wale direction to ensure uniform tension on the sensor. Alligator clips were attached to snap fasteners connected to the sensor, and measurements were performed with an MS technologies probe station (Tektronix, Beaverton, OR, USA) using the Keithley 4200-SCS system.

### 3.3. Bending Test

**Figure 2:**
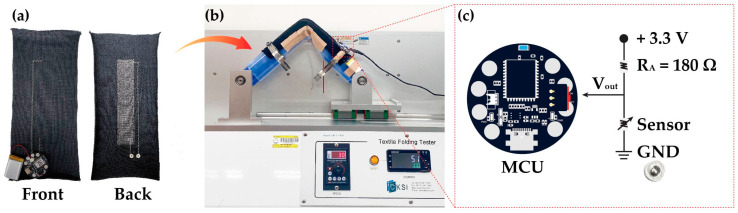
Preparation for bending test: (**a**) front and rear sides of knitted strain sensor connected to MCU; (**b**) E-textile flexing tester; and (**c**) circuit diagram for calculating sensor voltage.

Before starting the bending test, the sensors with the optimal yarn thickness and NP numbers for the best stitch patterns were selected based on the results of the stretching test. The bending angles were set to 60°, 90°, and 120° with reference to Cho et al. [36], and the bending rates were set to 10, 30, and 50 cycles per minute (cpm) to analyze the resistance change with bending rate. We evaluated the dependence of sensitivity, reproducibility, reliability, and responsiveness to the bending angle and bending rate to develop sensors optimized for joint motion monitoring. Figure 2a shows the knitted strain sensor used in the bending test. To measure the voltage change, a spring snap was fastened to connect the sensor to the voltage common collector and GND of the MCU. As shown in Figure 2b, a custom-made E-textile flexing tester (CKFT-T400, Netest, Hwasung-si, Republic of Korea) was employed to measure joint movement. Figure 2c presents a circuit diagram depicting a voltage of 3.3 V from an Arduino-based MCU (ESP 32-PICO-V3, Indifrog, Seongnam-si, Republic of Korea) being used for data collection. When connected to the Arduino program, 1 data item per 0.1 s was output to the serial monitor during 100 repeated sensing measurements. The MCU converts the deformation of the sensor to an output voltage ranging from 0 to 4095.

(2) $$V_{B}=\frac{output\left(sensor\;value\right)\times 3.3}{4095}\;V_{A}=3.3-V_{B}\;I_{A}=\frac{V_{A}}{R_{A}}\;R_{B}=\frac{V_{B}}{I_{A}}$$

The Arduino output voltage was converted to a resistance value using Equation (2); where $V_{B}$ is the sensor’s output voltage, $V_{in}$ is the input voltage (3.3 V), $V_{A}$ is the voltage across the fixed resistor, $R_{A}$ is the fixed 180 Ω resistor embedded in the MCU, and $R_{B}$ is the variable resistor of the strain sensor, which changes with strain. GF can be calculated by applying the measured resistance value to Equation (1):

(3) $$\varepsilon =\frac{∆R/R_{0}}{GF}$$

Using GF as a fixed constant, the material strain can be inversely estimated from the sensor’s resistance change and it can be calculated from Equation (3), where ε is the strain, $R_{0}$ is the initial resistance, ∆R is the resistance change due to strain, and GF is the gauge factor. 

## 4. Results and Discussion

### 4.1. Stretching Test Results

**Figure 3:**
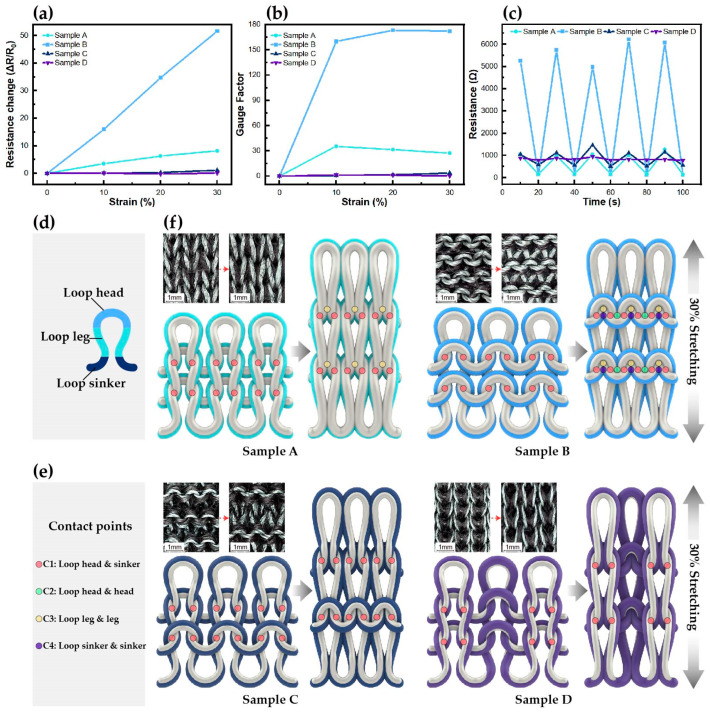
Variations in (**a**) resistance change rate and (**b**) GF with stretching; (**c**) results of five repeated stretching tests; (**d**) loop component; (**e**) contact points; (**f**) sensor surface variations and sensing mechanisms before and after stretching.

Figure 3a–c shows the resistance change measured while stretching the knitted strain sensors up to 30% in the wale direction. Sample A had the plain stitch with a conductive yarn located on the front, Sample B had the plain stitch with a conductive yarn located on the reverse side, Sample C had the purl stitch, and Sample D had the rib stitch. Figure 3a shows the rate of resistance change due to stretching for each sample. Sample B showed the best results, with a linearly increasing rate of resistance change. Figure 3b shows the variations in GF with stretching; for Sample B, GF increased to 143.68 at maximum stretching, which represents the best result. Both the aforementioned results indicate that the plain-patterned samples produced with the plating stitch were the best among the four samples. More specifically, the superior results of Sample B confirm that the plain-patterned reverse side with the plating stitch structure is the most suitable for detecting large deformation. Figure 3c shows the results of a test repeated five times, where the sensors were stretched up to 30% in the wale direction. Compared with the other stitch patterns, the plain stitch exhibited a relatively stable resistance behavior with stretching. Specifically, Sample B maintained a uniform shape with five bending state peaks despite exhibiting a high rate of resistance change; therefore, it can be considered suitable for joint motion monitoring applications that require repetitive sensing. These findings confirm that the plain stitch with a conductive yarn located on the reverse side offers excellent sensitivity and reproducibility.

#### Changes in Contact Points with Stretching

Based on the sensing mechanism, we explored the effect of the loop connection method on the number of contact points through photography and 3D modeling. As shown in Figure 3d, the loop consisted of three sections: the head, leg, and sinker; among these, the loop head and sinker in the wale direction were connected [37,38]. When the knit was stretched in the wale direction, the contact area and pressure between the head and the sinker increased, and the gap between the loops narrowed, thereby increasing the number of contact points. Thus, we defined four structurally generated contact points in this study, designated as C1–C4. As seen in Figure 3e, C1 represents the contact points between the heads and the sinkers, C2 represents the contact points between the heads themselves, C3 denotes the contact points between the legs themselves, and C4 denotes the contact points between the loop sinkers themselves. Figure 3f illustrates the surface changes and sensing mechanisms of the four sensors in the initial and 30% stretched states. The initial contact point for Sample A was C1, and after stretching, the contact points were C1 and C3. The initial contact point for Sample B was C1, and after stretching, contact occurred at C1, C2, C3, and C4. In Samples C and D, contact occurred only at C1, both before and after stretching. Thus, the plain stitch had relatively more contact points, and the largest number of contact points was observed in Sample B, which showed excellent performance in the stretching tests as well. The reason for the creation of more contacts in the plain stitch structure is that the loops are connected on the same location. In the rib and purl stitch, the front and back loops intersect to create a loose weave, whereas in the plain stitch, only the front loops are connected, creating a tight weave. Thus, even under the same deformation, the contact pressure becomes relatively high, and uniform shape deformation occurs. In addition, the yarn changes from a loop shape to a straight shape during stretching. The plain stitch is made with plating knitting techniques, making it easy to separate the nonconductive yarn and conductive yarn and form electronic paths. In other words, this loop connection method and knitting method evenly distribute the force at each contact point and facilitate electron flow, allowing the plain structure to exhibit higher sensitivity and excellent reproducibility to deformation. While Samples C and D had the same number of contact points after stretching, the sensor performance differed because nonconductive yarns intervened, and the contact area between the conductive yarns varied with the loop connection method. These findings confirm that contact points are created and changed during deformation depending on the loop connection method, thereby affecting sensor performance. Additionally, the plating stitch structure proved to be an excellent knitting method because it places loops uniformly in the front or back, reducing the intervention of nonconductive yarns and facilitating uniform shape deformation. Therefore, Sample B was considered the most suitable for joint motion monitoring.

### 4.2. Bending Test Results

Based on Sample B, six sensors were manufactured with different combinations of yarn thickness (1-ply and 2-ply) and NP numbers (12r, 13, and 14), which were selected through a stretching test. These sensors are designated as BX-Y, where X is the yarn thickness and Y is the NP number.

**Figure 4:**
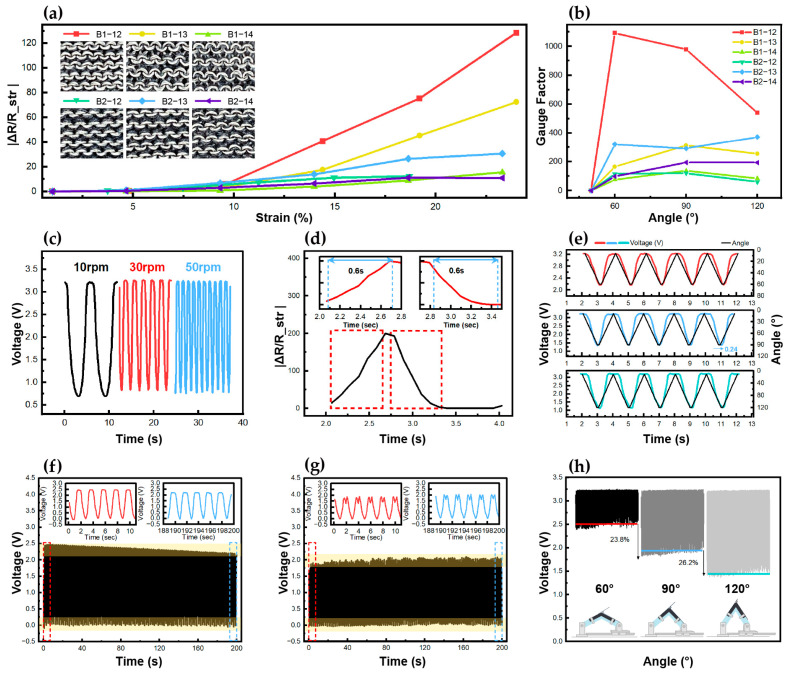
Bending test results: (**a**) 90° bending tests of six samples converted to strain rate; (**b**) GF results for 60°, 90°, and 120°; (**c**) response to bending rate changes in B1-12; (**d**) reaction time depending on bending rate for B1-12; (**e**) voltage change by angle for B1-12; 100-cycle repeated bending test of (**f**) B1-12 and (**g**) B2-12; and (**h**) voltage level according to bending state for B2-14.

Each sensor had a different stretch rate depending on the yarn thickness and NP numbers. The stretch rates of all the sensors by angle are summarized in Table 1. We conducted tests considering angles of 60°, 90°, and 120° and bending rates of 10 cpm, 30 cpm, and 50 cpm to examine the changes in resistance and voltage with angular deformation and the bending rate. The sensor performance was analyzed in terms of sensitivity, reproducibility, reliability, and responsiveness because the sensor must accurately detect continuous large deformations and fast joint movements in real time. Figure 4a presents the results of 60°, 90°, and 120° bending at 30 cpm, which were used to determine if the resistance increased proportionally with strain. It increased rapidly after the 10% stretch rate for the 1-ply sensors and after the 5% stretch rate for the 2-ply sensors. This is because using two nonconductive yarns reduces the space between the loops, allowing for faster contact between the conductive yarns. Figure 4b shows the results of 60°, 90°, and 120° bending at 30cpm based on which the GF for each angle was determined to evaluate sensitivity. For the 1-ply sensors, a higher NP number led to a weaker bonding force and fewer contact points between the loops, resulting in a lower GF. However, the 2-ply sensors showed the best results with an NP number of 13. Among the six sensors, B1-12 had the best GF values at 60° (1092), at 90° (978.7), and at 120° (540.2), whereas B2-12 showed the lowest GF values at 60° (115.2), at 90° (120.2), and at 120° (60.2). This sharp reduction in GF despite the same NP number was observed because the addition of a ply leads to an excessively high inter-loop contact pressure and a significant reduction in the inter-loop space, resulting in a substantial reduction in the stretch rate. This confirms that both the yarn thickness and NP numbers significantly affect GF; thus, appropriate NP numbers should be selected depending on the yarn thickness. Nevertheless, the GF values suggest that by adjusting the variables according to the magnitude of joint movement, all six sensors can be utilized for joint motion monitoring. Figure 4c presents the voltage changes measured in real time under bending rates of 10 cpm, 30 cpm, and 50 cpm for a bending angle of 90°; these results were used to evaluate the sensor’s response according to the bending rate. B1-12 achieved the best result, exhibiting a constant current regardless of the bending rate. All other sensors showed uniform voltage changes, without any significant effect from the yarn thickness and NP numbers. This indicates that the sensors can detect movement in real time regardless of the bending rate. Figure 4d presents the sensor’s reaction time for a bending rate of 30cpm and bending angle of 90°. The sensor must bend within 6 s at 10 cpm, 2 s at 30 cpm, and 1.2 s at 50 cpm. B1-12 achieved the best results in this regard, requiring 0.6 s to bend and 0.6 s to recover. Overall, the 1-ply sensors were more responsive than their 2-ply counterparts because fewer nonconductive yarns could intervene in the 1-ply. Nevertheless, all the sensors were found to be suitable for real-time joint motion monitoring because the movements were executed within an appropriate amount of time. Figure 4e presents the rates of change in the angle and voltage of the sensors for 60°, 90°, and 120° bending at 30 cpm; these results are used to verify the voltage change caused by angular deformation. B1-12 exhibited a maximum deviation of 0.24 s, and the voltage change closely matched the angle graph. While the other sensors showed slight differences depending on the yarn thickness and NP numbers, the responsiveness of all the sensors was excellent, with an error of less than 1 s. This verifies that all sensors can accurately measure joint movement for different angles regardless of the bending rate. Figure 4f,g present the 100-cycle repeated bending results of B1-12 and B2-12, respectively, considering 90° bending at 30 cpm, based on which the reproducibility of the sensors was evaluated. As the cycle is repeated, a drift phenomenon has been identified with a gradual increase in bending and recovery baselines across all sensors. These drift phenomena occur during stretching and recovery due to the inherent characteristics of common textiles and are affected by the properties of the material. Although elastic yarn is known to improve drift [39], nonconductive yarns composed of non-stretchable acrylic and wool were used in this study to accurately understand the structural changes and sensing mechanisms that occur in knitted strain sensors during deformation. Thus, we designed the sensors in a multilayered structure to improve mechanical stability and distribute pressure uniformly to mitigate drift. As a result of the analysis, it was confirmed that the drift phenomenon of B1-12 was more pronounced than that of B2-12, and the higher the nonconductive yarn ratio, the better the stability. The baseline of the B1-12 is changing due to drift, but it can be used as a sensor because the change shows a consistent rising pattern. This phenomenon could be further improved by adding elastic yarns and applying drift correction algorithms in the future. The double peak phenomenon in the recovery state is another characteristic of textile sensors, and this was particularly prominent in B2-12. As this sensor was relatively non-stretchable, it appears to have been impacted by the process of elasticity restoration [40]. However, because the double peak pattern was regular, the sensing function would not be compromised. Thus, all six sensors displayed excellent reproducibility regardless of the yarn thickness and NP numbers and could stably measure repetitive movements. Figure 4h presents the voltage levels measured under 60°, 90°, and 120° bending at 30 cpm. B2-14 showed the best results, with its average bending state value increasing by 23.8% from 60° to 90° and by 26.2% from 90° to 120°. The increase was similar for each angle, and the responsiveness at each angle was the highest as well. The average bending state value of B2-12 increased by 16% from 60° to 90° and by 3.9% from 90° to 120°, which represents the lowest increase among all the sensors. Nevertheless, the voltage values of all the sensors could be distinguished by angle regardless of yarn thickness and NP numbers; a similar pattern was observed when the bending rate was altered. This indicates that all the sensors can detect joint motion at different angles. Overall, these results suggest that all the sensors possess excellent sensitivity, reproducibility, reliability, and responsiveness and that the plain-patterned plating stitch structure with conductive yarns on the reverse side is ideal for joint motion monitoring.

**Figure 5:**
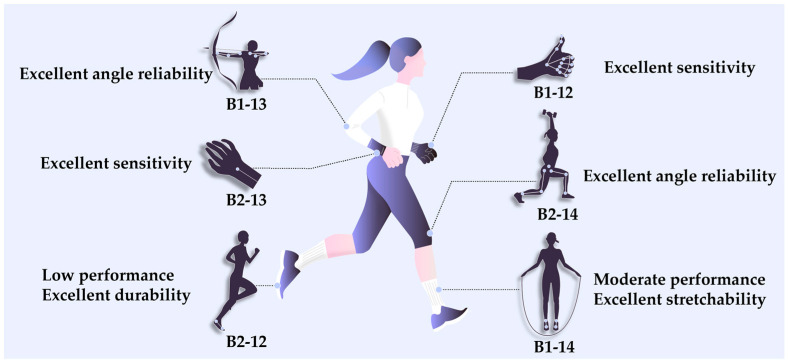
Application prospects of the sensors developed in this study.

Based on the results obtained, Figure 5 summarizes the smart fashion products for which the proposed sensors are considered suitable. For example, the 1-ply sensors are more elastic, thinner, and lighter than the 2-ply sensors, while the sensors with larger NP numbers achieve better strain rates. Therefore, these sensors can be used in products that require high elasticity. For example, because B1-12 exhibits the highest sensitivity and achieves excellent results in terms of the other parameters as well, it can monitor small to large movements. Therefore, it can be used in smart gloves for motion recognition at finger joints. Similarly, B1-13 provided relatively reliable measurements of angles of 0–90°, achieving the best results among the 1-ply sensors. Therefore, it can be applied to the elbow and used for archery sportswear, which requires precise angle measurements. Additionally, B1-14 is the most stretchable sensor, but it did not achieve the best performance in all aspects. Therefore, it can be worn on the ankle in the form of a band to count the number of movements such as jumps when using a jumping rope. The 2-ply sensors are less elastic than that of 1-ply sensors but are more durable; moreover, their elasticity can be increased by increasing the NP number. For example, B2-12, which has the lowest elasticity and achieved relatively low performance, can be integrated into shoes to track the number of steps. Additionally, B2-13 has the best sensitivity among the 2-ply sensors and responds to relatively small stretches. Therefore, it can be incorporated into injury-prevention bands that monitor small movements of the wrist. Finally, as B2-14 offers excellent angle-recognition performance, it can be used in sportswear for applications where angles are important, such as squats and lunges.

## 5. Conclusions

In this study, we analyzed the effects of the stitch pattern, yarn thickness, and NP number on the performance of knitted strain sensors to develop a sensor optimized for monitoring joint motion. To determine the optimal stitch pattern, we conducted a stretching test on basic patterns of weft-knitting fabrics; the plain stitch with conductive yarns located on the reverse side yielded the best results. It had a GF of 143.6 at maximum stretching, which was at least five times that of the other patterns, and exhibited stable resistance changes even after repeated sensing. The plating stitch structure also reduced the involvement of nonconductive yarns during stretching, facilitating uniform shape deformation. To ensure optimal performance for joint monitoring, six sensors were developed with different combinations of yarn thickness (1-ply and 2-ply) and NP numbers (12, 13, and 14) using the plain stitch with conductive yarns located on the reverse side. Bending tests conducted to examine sensitivity, reproducibility, reliability, and responsiveness based on the bending angle and bending rate revealed differences in performance between the sensors. In particular, the GF ranged from 60.2 to 1092 across all sensors, indicating a large variation in sensitivity with the yarn thickness and NP numbers. The other performance parameters were less affected by the yarn thickness and NP numbers and indicated excellent results overall. These findings suggest that all the developed sensors can be used for joint motion monitoring, with the specific purpose depending on the adjustable GF value. Therefore, our study is significant, as it validates the structural superiority of the plain stitch with conductive yarns located on the reverse side and introduces an optimized plating stitch technology to enhance the performance of knitted strain sensors. Additionally, as the developed sensors are suitable for all areas where joint movement occurs, they are expected to be applicable in various smart fashion products. However, in-depth studies of their long-term stability are essential for practical applications of sensors. In addition, various environmental factors such as temperature and humidity and durability factors such as washing and abrasion are also important considerations to maintain performance in everyday-use environments. Since this study was limited to nonconductive yarns, we intend to investigate the long-term stability of the plating stitch structure sensor in more detail using elastic yarns in future studies. This study will contribute to accelerating the development of strain sensors and increasing their applicability in actual industrial fields.

## Figures and Tables

**Table 1:** Stretch rates of sensors by angle.

Angle (°)	Stretch Rate (%)					
Sensor No.	B1-12	B1-13	B1-14	B2-12	B2-13	B2-14
60	15.3	15.3	16.7	15.3	16.7	16.7
90	24	24	23.3	18.7	23.3	23.3
120	31.3	33.3	30	26.7	30	33.3

## Data Availability

Data are contained within the article.
